# The relationship between subjective well-being and food: a qualitative study based on children’s perspectives

**DOI:** 10.1080/17482631.2023.2189218

**Published:** 2023-03-09

**Authors:** Cristina Vaqué-Crusellas, Mònica González-Carrasco, Ferran Casas

**Affiliations:** a Department of Social Sciences and Community Health, Centre for Health and Social Care Research (CESS), University of Vic – Central University of Catalonia, Vic, Spain; b Department of Psychology, University of Girona, Girona, Spain; c Faculty of Education and Social Sciences, Department of Psychology, University Andrés Bello, Santiago, Chile

**Keywords:** Children, well-being, food, food well-being, satisfaction with food, subjective well-being

## Abstract

**Purpose:**

Despite the lack of consensus regarding which life satisfaction domains should be included in the study of children’s subjective well-being (SWB), some domains are frequently considered, such as satisfaction with health. However, some others, such as satisfaction with food, are barely taken into account, despite the impact eating habits have on children’s health and well-being. We adopt a qualitative approach to explore the role food plays in children’s SWB, providing for a more in-depth analysis of children’s perceptions and evaluations on a still insufficiently known domain of life satisfaction.

**Method:**

Sixteen discussion groups were held with 112 Spanish students (10–12 years old) from six schools. The transcripts were analy sed and themes reflecting the key concepts were defined using reflexive thematic analysis.

**Results:**

Five themes emerged from the children’s discourses on the relationship between food and SWB: health, pleasure, emotions, commensality—i.e., eating together—and food-empowerment—thus offering new insights from children’s perspectives.

**Conclusion:**

Almost all of the participants established a relationship between their SWB and their eating behaviour, meaning that, within the challenges facing public health, SWB must be taken into account when promoting healthy eating programmes for children. Also, group discussion is found to be a very powerful tool for exploring topics with subjective connotations among child populations.

## Introduction

It is considered that subjective well-being constitutes the articulation of a cognitive process (satisfaction judgements with life considered globally, and with different specific life domains, such as, among others, health, school, family, friends, economic aspects and relationships with others) and two affective processes (positive affects and negative affects) (Cummins & Cahill, 2000). It follows that a person who experiences more positive emotional experiences throughout life (e.g., cheerful, energetic, in a good mood, etc.) than negative experiences (fear, anger, sadness, etc.) will probably perceive life more satisfactorily.

Although subjective well-being in adults has been widely studied (Cummins & Nistico, 2002; Diener et al., 2003; Michalos, 2008, among many others), interest in knowing how it works among the child population did not emerge until the late 1990s, after children’s rights had been recognized as human rights (United Nations Convention on the Rights of the Child, 1989). This recognition established the consideration of children as active subjects in defining their well-being, understanding that it is through the experiences, meanings and opinions of children themselves that the key areas of their well-being and quality of life will be known (Casas, 2010; Fattore et al., 2007; Sorbring & Kuczynski, 2018).

Although there is no international consensus on which areas of satisfaction with life should be studied in order to determine people’s subjective well-being, some authors do agree on some domains, as is the case with health (Bradshaw et al., 2011; Casas et al., 2007; Rees & Main, 2015; Tomyn & Cummins, 2010; Vujčić et al., 2019), while others, such as food, are barely taken into account. That being said, some authors have begun to develop and study the concept of “Food well-being (FWB)”, defined as a positive psychological, physical, emotional and social relationship with food at both the individual and societal levels. They highlight the important relationship between food and subjective well-being in contributing to a better understanding of consumer food choices (Ares et al., 2014; Block et al., 2011; Hémar-Nicolas & Ezan, 2019; Sobal et al., 2006).

If we understand health as a state of complete physical, mental and social well-being, and not only as the absence of disease (WHO, 1948), it makes sense to think that subjective well-being is a determining component of health and that its study can contribute to knowing and designing better health promotion strategies, also among the child population.

In this study, we adopt a qualitative approach to explore the role of food in subjective well-being based on children’s perceptions, assessments and opinions in relation to this issue, following the approach of social sciences, given the premise that food is one of the main determinants of health and a key piece in the notable challenges facing public health globally.

WHO data (2018) show that the prevalence of chronic non-communicable diseases is constantly on the increase (obesity, diabetes, cardiovascular diseases, etc.). IT IS ALSO REINFORCED BY NCD RISK FACTOR COLLABORATION NETWORK (2022). Many interventions have been developed to improve these conditions, based on promoting knowledge and skills to reduce weight and the sedentary lifestyle and adopt a healthier diet by working on aspects of behaviour (self-efficacy, expectations, subjective norms), as well as actions to promote healthier environments. Few of these strategies focus on exploring whether food and the adoption of specific dietary patterns are associated with people’s subjective well-being, linked to beliefs, opinions and emotions that are experienced individually but socially constructed, through interaction with others. These beliefs and emotions can also be expressed in relation to the foods and eating habits that characterize a particular social group. Such an association can have important consequences, because if healthy practices are more unpleasant than pleasurable, then they may not be established and will therefore not be maintained, because they will not be satisfactory to the individual. On the other hand, if people do see health as an important goal, then food can help them devote more effort to deploying healthy eating habits and lifestyles; otherwise, the adoption of certain food practices will be much more difficult.

That is why studying the relationship between subjective well-being and eating habits in children can provide important information that helps to have a positive impact on their healthy development, not only during childhood and adolescence, but also in adulthood.

Food is an area where aspects of pleasure (hedonism) and self-regulation (eudemonism) come together, constituting a source of satisfaction or dissatisfaction in people’s lives, this contributing to reconcile two traditions that have traditionally been addressed separately in the study of well-being (Ryan & Deci, 2001; Strelhow et al., 2020). A consequence of the above is that most studies designed to study well-being employ measures that do so from a hedonic perspective, and do not include measures from the eudemonic side (Sawatzky et al., 2009). However, the population, and even the youngest members of it, has increasingly more information regarding the impact of eating habits and lifestyles in general on health (Haines et al., 2019; NCD risk factor collaboration, Vaqué-Crusellas et al., 2015; World Health Organization, 2018), raising awareness regarding these issues.

In the review of the literature on subjective well-being in children conducted by Pollard and Lee (2003), which included a total of 175 publications, the authors found that, despite the great disparity in indicators for measuring children’s well-being, health promotion and nutrition are considered positive elements in this regard. However, the research analysed did not delve further into elements that lead to health and nutrition being important aspects of children’s well-being, and more research is needed to determine this.

The study by Lindberg and Swanberg in 2006 focused on the subjective well-being of 807 12-year-old boys and girls in Sweden, highlighting that issues related to health, nutrition and physical activity were predictive elements of the subjective well-being of the children who participated in the study, along with other factors such as interpersonal relationships (parents, teachers and friends), anxiety, depression and somatic symptoms (headache, stomach ache, back pain, difficulty sleeping). These results point to the existence of an association between the two concepts (food and subjective well-being), while indicating that the relationship can be positive and increase subjective well-being. More studies are required to determine whether the relationship between these two concepts can also be negative, and therefore diminish the well-being of boys and girls.

Chang and Nayga (2010) explored the relationship between fast food consumption and sugary drinks and the happiness of a sample of 2,366 participants aged 2 to 12 years old from Taiwan. They measured unhappiness by asking parents how often the child was “unhappy”, “sad” or “depressed”. The results showed that those who consumed fast food and sugary drinks displayed a lower significant positive correlation with being unhappy than boys and girls who did not consume fast food and sugary drinks, who displayed a higher, though still low, correlation with being unhappy. The authors highlighted the interest of these results, especially for food policies aimed at combatting childhood obesity, which usually focus on aspects of physical health and pay less attention to aspects of well-being. They also concluded that in order to reduce childhood obesity, programmes need to propose strategies to compensate for the potential reduction in children’s happiness due to their not consuming certain products that subjectively generate their well-being.

Vaqué et al. (2015) used an extended version of the Personal Well-being Index-School Children version (PWI-SC) by Cummins and Lau (2005), adding an item related to satisfaction with food. They verified the psychometric properties of the new index, and tested it on a sample of 371 children aged 10–12 in Spain. The results showed that the inclusion of a domain on satisfaction with food as a proposed new indicator to study subjective well-being in this age group contributed to the PWI-SC with unique variance (6.7%), displaying an increase of 3.6% in shared variance.

To explain levels of SWB in theoretical terms, measured by means of the PWI-SC, the authors hypothesize the existence of a homeostatically genetic mechanism, analogous to how the body controls its biological systems, such as blood pressure and heart rate. This mechanism would be responsible for relatively small variations in SWB levels among people of the same culture (unless protective factors have failed to prevent levels from decreasing) (Cummins, 2014). In relation to satisfaction with food, this would mean that certain life or personal circumstances might lead some children to assess this life domain very negatively, but that in most cases these levels would gradually recover at some point.

Arvidsson et al. (2017) investigated the association between children’s adherence to healthy dietary guidelines and their well-being. A sample of 7,675 children aged 2 to 9 were studied. A higher Healthy Dietary Adherence (HDA) score at baseline was associated with better self-esteem and fewer emotional and peer problems. Conversely, better self-esteem was associated with higher HDA score two years later. These findings suggested a bidirectional relationship between diet quality and self-esteem. Additionally, higher adherence to healthy dietary guidelines at baseline was associated with fewer emotional and peer problems at follow-up, regardless of children’s weight status.

The study by Hayhoe et al. (2021) investigated the association between dietary choices and mental well-being among schoolchildren. Data pertained to 1,253 primary school children in Norfolk. The findings indicated that type of breakfast or lunch was associated with significant differences in well-being scores. The authors observed that, compared with children consuming a conventional type of breakfast, those not eating any breakfast had lower mean well-being scores, although no significant association was found with fruit and vegetable intake. Despite this, the authors still concluded that public health strategies to optimize children’s mental well-being should include the promotion of good nutrition.

Brennan et al. (2021) studied the impact of a food intervention conducted in 15 primary schools in Ireland (6–11 year-olds) with the aim of altering the school’s food environment, offering experiences related to agriculture, cooking and food nutrition. Improvements in childhood emotional and behavioural well-being, dietary intake, knowledge about food, cooking skills and willingness to try new foods were found to be associated with this food intervention.

In the present study, we have explored well-being based on children’s own assessments, and not through the opinions of other people (proxies), even if they are close to the children. We adopted a qualitative approach to gain a broader view that emerged from a framed conversation on the topic being studied. The reasoning or reflection required of the children led to the decision that this research should not be carried out with very young children. Several publications (Adams et al., 2016; Camfield et al., 2009; Fattore et al., 2016) have used qualitative data collection and analysis techniques to learn more about the great diversity of children’s opinions and assessments in relation to their well-being. The qualitative approach provides in-depth information on the subject of study, complementing aspects traditionally investigated through questionnaires, which call for answers on pre-established dimensions of the studied construct.

**Figure 1.**
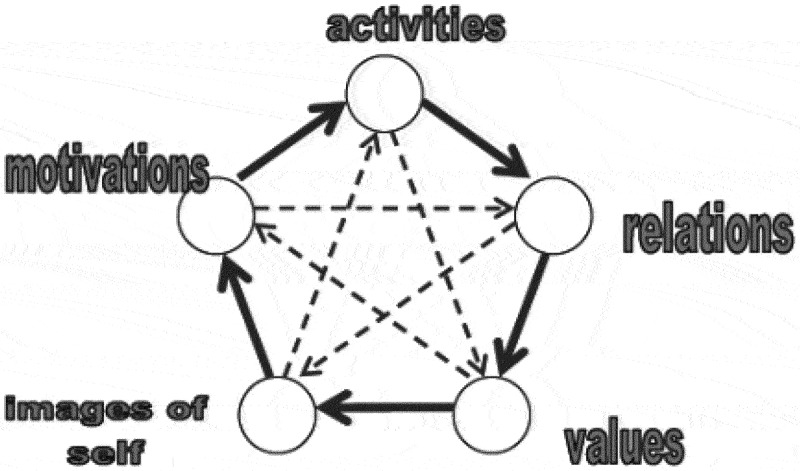
The actor’s system theory (Stoecklin, 2013).

More specifically, the theoretical framework adopted within this qualitative research is Stoecklin’s (2021, 2014, 2013) *actor’s system theory*. According to this model, despite it representing a notable step forward within childhood studies, it is not sufficient to consider children as social actors, since their agency depends on dynamic relationships with other children and adults and the specific contexts in which these interactions take place. Consequently, the actor’s system theory represents discursive horizons that are commonly used by social actors when describing their daily experience, which are activities, motivations, relations, values and images of self (Figure 1). Social actors frame their lived experience within these “transactional horizons”, which can be defined as symbolic landscapes channelling social interactions. Although this theory has been applied to conduct in-depth research into children’s well-being on a global scale, we believe it can also be used to understand the connections that might exist between the particular case of subjective well-being and food from children’s perspectives.

The plain arrows symbolize a habilitating force and the dotted arrows a constraining force

There are several qualitative techniques applied to the study of subjective well-being and used especially with children. Discussion groups are widely used in social research to discuss a specific topic, with the aim of drawing on the complex personal experiences, beliefs, perceptions and attitudes of the participants through a moderated interaction (Nyumba et al., 2018). This technique provides an outstanding contribution for understanding how children perceive themselves and the world around them, awarding them a central role. Our definition of a discussion group with children and adolescents is as follows: *a group dynamic in which participants assume they are experts on some topic and advise adults from their own perspective following discussion with one another about what adults should do or understand, while the adults involved listen to them and only ask for clarifications about the meaning of what they say* (González-Carrasco et al., 2021, p. 154).

We believe this approach allows us to explore in greater depth children’s understanding about the extent to which they consider food an important element in their subjective well-being.

## Materials and methods

### Design

An exploratory design was adopted for this qualitative study, this representing an ideal approach when few data are available, since researchers can then take a primarily inductive approach to explore a broad research question (Rendle et al., 2019).

### Participants

Altogether, 112 students from six schools in the county of Osona (Catalonia, north-eastern Spain) were recruited to participate in 16 discussion groups. These schools were recruited by means of intentional sampling, looking for heterogeneity in terms of their location (66.7% urban and 33.3% rural schools) and type of school (66.7% state schools and 33.3% mixed funding schools). The discussion groups were organized based on the participants’ gender (four females-only, four males-only, and eight mixed groups) and age (eight with children aged 10–11, and eight with children aged 11–12, see Table I). These ages correspond to the last two years of primary education within the Spanish education system and were chosen because they are appropriate for reflection and discussion on the issues raised.

**Table I.** Characteristics of the sample.

	Type of school	Location	Number of discussion groups	Age	Gender	Number of participants
School 1	State	Urban school	1	10–11 years	Mixed	8
School 2	State	Urban school	2	10–11 years 11–12 years	Mixed Mixed	8 8
School 3	State	Rural school	6	10–11 years 11–12 years 10–11 years 11–12 years 10–11 years 11–12 years	Female Male Female Male Mixed Mixed	6 6 6 6 8 8
School 4	State	Rural school	2	10–11 years 11–12 years	Mixed Mixed	8 8
School 5	Mixed funding	Urban school	4	10–11 years 11–12 years 10–11 years 11–12 years	Female Male Female Male	6 6 6 6
School 6	Mixed funding	Urban school	1	11–12 years	Mixed	8
Total			16			112

The size of the groups was expected to be between six and ten participants in order to allow everyone the opportunity to speak, ensure maximum fluency in the conversation and encourage a comfortable space to express opinions without the risk of establishing discussion subgroups. Students with cognitive impairment were not included. The number of discussion groups was determined based on the saturation and richness of the information obtained.

### Procedure

The aim of the discussion groups was to explore the children’s understanding of the relationship between subjective well-being and food, if any. A panel comprised of four academic experts in the areas of social psychology and paediatric nutrition was consulted to review and refine the preliminary questions included in the discussion group, the aim being to improve understanding, clarity and focus with regard to these questions. Subsequently, a pilot discussion group was conducted with children (*n* = 7, age between 10–12 years old) with the purpose of testing and improving the questions. After changes were made, the questions were returned to the expert panel for further review before starting the study. The panel verified that the children’s answers addressed the defined objective.

Using the final instrument obtained, we began the discussion by talking about food in general, and then what subjective well-being meant to the participants. We decided to ask this without making explicit reference to the construct of “subjective well-being” because it is not part of the participants’ usual vocabulary. The results shown in the article relate to the opinions that emerged openly and spontaneously when posing the first questions about food and well-being. They were complemented by opinions obtained by explicitly asking the question linking the two concepts.

The time needed to conduct each discussion group was around 1.5 hours, and all of them took place in the school setting under the guidance of the same researcher.

### Ethical considerations

Prior to the start of the sessions, informed consent was obtained from the participants, parents, as was permission from the school, in order to be able to record the conversations and ensure that the children’s participation in the study was voluntary. The participating schools received authorization from the families to involve the children in different activities throughout the school year. When the time comes, the school informs the families of each proposal, and they again have the opportunity to refuse their child’s participation. Table I describes the characteristics of the sample.

### Data analysis

The children’s statements were analysed by means of thematic analysis (Braun & Clarke, 2006), which began with the careful transcription and reading of each discussion by the researchers in order to familiarize themselves with the information obtained. Subsequently, a thematic and open encoding process was undertaken in order to organize and segment the text. And finally, the codes were grouped into topics and subtopics with the aim of focusing, simplifying and abstracting the most relevant data by grouping them into significant topics according to the aim of the study.

Two experts reviewed the proposed categories—both were senior psychologists. One was working as a professor at the University of Girona, and the other as an independent researcher. They had extensive experience in the field of social and health psychology, conducting both quantitative and qualitative data analysis and incorporating intersectionality into their work. Interjudge analysis (Neuendorf, 2002) was used in order to increase reliability and reach a consensus in the number of categories proposed and their interpretation. The NVivo program was used for the analysis.

Although the information provided was analysed according to participants’ age and gender, the reflections provided were found to be common, and they are therefore presented together.

## Results

The results presented in this article are related to the main topic debated in the discussions groups, namely the relationship between food and well-being, given that a clear relationship was observed between these two concepts for most of the participants. They related food and subjective well-being either in a *general* way (without specifying the reasons for this link), or for the reasons of *health* and *preventing illness*, *growth* and even *to be happy*.

L. - “if you eat well and we grow big and strong from all the food, and we don’t get diseases. I sometimes talk to my mother about this issue and of course, it’s important to eat fruit and vegetables to be healthy, and not have health problems”
P. - “because you eat healthy things that have iron and energy and you will be better, because if you never eat anything with energy you stay skinny and you don’t grow so tall”

Related to food **pleasure**, most participants indicated clear satisfaction when they ate what they liked and thought was good (foods that tasted good and smelled good), such as soft drinks, chocolate, spaghetti, ice cream and chips, among others, mostly unhealthy food. As for **emotions**, they explained that eating foods they like improves their well-being because they feel good, in some cases specifying that they feel happy, content and good in themselves.

J. - “I love to eat tasty things, like when I have a snack, for example, eating a chocolate croissant is delicious and makes me feel good”
M. - “I’m happy when I get home for lunch and see they’ve cooked what I like and not vegetables or fish, which are horrible”

In some cases, the feeling of vitality was also identified. They noticed or believed that once they had eaten they had a lot of energy to do any activity.

C. - “When I eat, I notice that I have energy, and then I feel like doing things because I’m not tired and I feel good”.
  Only in the case of one girl did she have the opposite feeling, as she explained that she felt guilty after eating “unhealthy” food.

Faced with responses regarding how they felt when they ate foods they did not like, all participants explained that it made them feel worse. This assessment evoked a wide variety of negative emotions, including, among others, feeling bad, sad, angry, uncomfortable and upset. Three participants reported that they lost energy, were very tired and had no strength to do anything.

O. - “it’s horrible to sit at the table and have to eat something you don’t like, like chickpeas boiled with vegetables. They’re disgusting and they stink. They make me eat it and I get very angry because I don’t like them at all. I hate them”
E. - “it’s annoying when you can’t choose your lunch because I’m happy in class, then I leave class very hungry, but when I see vegetable puree, ‘urrggh’, …, I get sad and feel bad because I have to eat it even if I don’t like it”
S. - “sometimes I really want to eat something but then when I see it’s something I don’t like, it makes me angry, because, for example, I don’t like salad and when I have to eat that my mood changes”

Another reaction found in their responses related to **health**, was the appearance of physical discomfort. They often described symptoms such as abdominal pain and vomiting as a result of eating things they did not like. Unlike what happened to them when they had food that they very much liked in front of them, in this case they lost the desire to eat and did not eat as much as they liked. They were no longer hungry when they knew they had to eat foods they would not be able to enjoy, while they were also no longer hungry when they knew they could do some other more desirable activity such as playing.

Table II summarizes the participants’ observations in relation to how they felt when they ate (Table II), with more opinions being observed in relation to negative feelings than to positive experiences, showing just how connected **emotions** are to food consumption and well-being from the children’s perspective.

**Table II.** Topics and subtopics related to the sensations produced by food.

Topics	Eating foods they like	Eating foods they don’t like
Sub-topics	Feeling good Happiness Feel vitality Wanting to eat Feeling good in themselves Feeling guilty	Feeling physically ill Thinking of strategies for not eating Feeling bad Losing appetite and energy Being upset Enraged Being sad Feeling uncomfortable

Some of the participants explained that when they had to eat things they did not like, they looked for multiple ways to avoid it. Sometimes they were allowed not to eat it, while at other times they had to deal with it by eating as little as possible. They would draw out the meal, eating very little, either because they did not finish it or they asked for only a little, they combined foods they liked with others they did not like so much, or they simply did not eat it, throwing it away or giving it to someone else or a pet:

B. - “I always cheat, for example with fish or something, like yesterday, my mother was working and my grandmother came, there was fish and my grandmother came to the kitchen and I still had two fish left, and I said to my brother: Do you want some? And he said yes, and I gave him a fish, and then my grandmother came, and I asked, ‘Do I still have to eat this one?’ And she said ‘No! It’s okay’, and I didn’t eat it.”
P. - “if I don’t like or don’t fancy something, and they don’t see me at home, I throw some to the dog and he eats it. Then my parents tell me, well, you’ve already eaten some, you can leave the rest”
J. - “if they force me to eat something I don’t like and don’t want, I imagine it’s a food I like more, like peaches, or I see if I can go to grandma’s house, which is next door, and she makes me what I want”

In addition, and in connection with child **empowerment** and the **context where food is consumed**, we observed a clear desire for them to choose what they wanted (both the type of food and the quantity) and in different contexts (school, at home or in restaurants), as well as not to be told off or forced to eat. A total of only four boys and girls explained that they sometimes accepted having to eat what they did not like because they valued the benefits it could bring them for their health, and understood it therefore did not have any negative effects on their well-being.

L. - “I don’t like vegetables or fruit or all the foods that at home and at school they tell us are healthy, but because we need them to be healthy, I eat them and nothing happens”.
B. - “parents do what they have to do to make sure we eat well so we won’t have problems, and they have to educate us, otherwise when we grow up we’ll just eat pizza, pizza and more pizza. Now I know that I have to eat everything and I’m happier eating it”

On two other occasions, the reasons for this acceptance were not specified, as it was only stated that they ate it anyway, showing compliance with what they were given to eat.

Eating in a place where they felt comfortable (not eating in a cold place, eating in open spaces such as in the mountains, or eating at grandma’s house), as well as feeling good about the people they shared their meals with, also made them feel at ease when eating:

P. - “I’d rather eat vegetables than be with people I don’t like.”
L. - “I don’t care if they make me eat a variety of food, but I would be very angry if I could never eat with someone I love very much”

However, in four cases, contrary explanations were also observed. In these instances, it was argued—though without much justification—that food had nothing to do with personal well-being, thus rejecting a relationship between food and well-being on the basis that other things influenced living well.

Generally speaking, however, there was an evident desire to not suffer while eating, but rather to feel at ease and have a good time eating so as to feel better and have a good level of well-being.

## Discussion

The aim of this study was to explore the extent to which food is an important element in children’s well-being, departing from the fact that “*it is central to know how children construct meaning around everyday lives* [food in this case] *and how the process of meaning meaning-making mediates their relationship with social reality*” [interactions with other people around food and its consumption for this study] (Stoecklin, 2021, p. 72).

The 10- to 12-year-old boys and girls participating in this study gave multidimensional arguments when referring to this relationship by observing that they virtually all related food to their well-being. This relationship refers mainly to five themes: health, pleasure, emotions, context (commensality, i.e., eating together), and children’s empowerment with respect to decisions related to food. Some of these issues that explained the participants’ well-being also emerged in a study conducted by Haines et al. (2019) aimed at understanding the opinion of French children aged 6–11 years regarding food well-being. Other researchers have observed this relationship in previous studies (Arvidsson et al., 2017; Brennan et al., 2021; Hayhoe et al., 2021; Lindberg & Swanberg, 2006; Pollard & Lee, 2003). This relationship highlights participants’ awareness of the importance of food in their lives, beyond its clear biological function. Only four of the 112 children who participated stated—though without much justification—that food had nothing to do with their personal well-being, meaning we would need to continue investigating which dimensions explain their well-being. Perhaps for these four children, food is not a priority in their lives and therefore not within the things they consider important to do (this referring to *activities* within the actor’s system theory), or they are good eaters and food does not cause them any rejection or negative emotions (food is probably not part of their *images of self*). Another explanation could be that their eating habits fit their expectations and needs, so they do not have any wishes in relation to this (*motivations*). These findings provide insights that can be used to develop strategies for promoting healthy dietary habits and increasing well-being by targeting different needs. Most of the participants associate these two concepts, suggesting that different strategies than the one currently used should be developed to promote food habits within children’s well-being.

It is likely that no relevant differences are observed by age because the age range studied was very limited (2–3 years) and the participants were not yet teenagers. In contrast with the qualitative study we present here, quantitative studies on nutrition and well-being reveal gender differences, with adolescent girls performing worse, probably due to the fact that girls tend to score higher in negative affect and are more affected by a negative perception of weight. In light of this, well-being and obesity prevention programmes should consider age and gender differences (Casas et al., 2007; Gaspar et al., 2017).

Understanding subjective well-being as a multidimensional assessment that reflects the combination of a cognitive process (satisfaction with life considered globally and also with different specific life domains: among others, health, school, family, friends and relationships with others), and two affective processes (the presence of positive affect and absence of negative affect), we can state that for most participants in this study, food is more present in the affective than in the cognitive dimension of subjective well-being. However, some participants also discussed the impact of what they eat on their growth and health. This highlights the awareness of the children who participated in the study regarding the relationship between food and health, which supports the need to improve eating patterns, especially among the child population, due to their protective effect for the development of future pathologies, as well as ensuring proper growth (World Health Organization, 2018).

According to the *relations* dimension within the actor’s system theory, the context in which children eat, as well as the people with whom they share the act of eating, are other relevant elements to take into account when promoting child well-being, given the influence that participants in this study awarded to the fact of feeling good when they are in quiet, pleasant environments and together with people they feel comfortable with. The negative effect on the participants’ well-being of not having the opportunity to decide on their own diet was also very evident. Studies by Hémar-Nicolas and Ezan (2019) and Van der Kaap-Deeder et al. (2017) have also shown this to be a significant issue for child well-being. Stoecklin and Fattore (2018) highlighted that agency is not something the child possesses but the product of a relational dynamic between social actors in specific contexts. In respect of this, the quotes of the participating children provide examples of the spaces in which food consumption takes place in their everyday lives, the different people who intervene in these spaces and even their capacity to “negotiate” with adult actors about what and how much to eat.

Participants showed a high interest in aspects related to food by highlighting the importance of food in their lives. Although we do not have data to state this categorically, the authors believe that this high interest could be the result of the numerous interventions in health education carried out in Spain in recent years (through studies on food promotion, the implementation of food issues on school curricula, the appeal of food transmitted to them by gastronomy, among other aspects). In terms of the actor’s system theory, this interest could also be interpreted as a mode of action, that is, “a typical way of acting according to dominant transactional horizons that link concrete items of perceived reality” (Stoecklin, 2018, p. 561).

All of the above elements may have laid the foundations for children to be more aware of food from an early age and reflect on it in a positive way, indicating a responsibility towards their health and self-control when it comes to food.

Most of the participants explained that eating good food (according to their tastes) benefits their well-being and makes them happy. This reasoning refers to the hedonic assessment of food aspects. Brillat-Savarin (1999) emphasized the importance of pleasure with food at any age, not exclusively in adulthood: “pleasure at the table is important for any age, class, nation and era. It can be combined with all other pleasures, and it survives all else to comfort us for the loss of others”. When people enjoy their food, it means that they are eating something they like, whether it is the taste, the aroma, the texture, the colours, the environment or the company they are in (Landry et al., 2018). The pleasure of eating also depends on a range of internal aspects, including motivational, cognitive and behavioural factors (Macht et al., 2005).

Only in one situation did a girl express a sense of guilt over eating something she likes. Negative emotions were also common when participants eat foods they do not like. Some explained that they get sad, angry, or even somatize the situation and suffer physical pain or discomfort, which negatively affects their well-being. Interestingly, in the face of this feeling of discomfort, some participants develop strategies to avoid it. This finding is important for health promotion framed from a hedonic and a salutogenic perspective (Antonovsky, 1979; Diener, 1984), through exploring alternative strategies such as the use of eating for pleasure to promote healthy eating and children’s well-being. Landry et al. (2018) voiced their support for this approach through a more holistic vision of the role of food in human well-being, rather than in physical health alone. We agree with their argument that nutritional policies should incorporate eating for pleasure and the enjoyment of food to promote healthy eating strategies.

The (positive and negative) associations highlighted by the participants also lead us to observe the same influence that food exerts on the nature of people’s emotional state in childhood as those noted in samples of young people and adults. These emotions are, among others, joy, satisfaction, sadness and anger (Ares et al., 2015; Bisogni et al., 2002; Desmet & Schifferstein, 2008; Hémar-Nicolas & Ezan, 2019; Macht et al., 2003), also showing that food does not affect children’s well-being only in nutritional terms.

This link between food and emotion can be used to guide strategies for promoting healthy eating in childhood, including fostering positive feelings when eating in order for children to associate food with a basic and enjoyable element in their lives. Paying more attention to children during meals to prevent them from throwing away food, and justifying the usefulness of following a balanced diet with a combination of foods that are not so much to their liking, can reduce the situations of physical and mental discomfort that some suffer and minimize feelings of anger or sadness.

One of the limitations of the present study is that the sample was formed of schools that expressed an interest in participating. It is therefore unknown whether non-collaborating schools had important differential characteristics with respect to the variables covered in this article. In the future, it would be good to collect data in a way that would increase the number of participating schools and allow for a longitudinal study combined with participant observation. It is worth noting that initially advising the discussion groups that the context of the talk was food, even if only through some very general instructions, may have resulted in the participants making much more extensive and in-depth reflections on the issues studied.

They generally felt free to express their opinions, and explicitly expressed satisfaction with being able to give their views. A friendly and familiar atmosphere was established, although on one occasion the group of students felt uncomfortable and unmotivated. The interview may have been badly timed in this case, as they were tired, and we did not get the project presentation right or clarify enough the importance of their participation.

As strengths of the study, we would emphasize that subjects assessing their own well-being allows us to ascertain accurate information regarding their beliefs and perceptions by providing important data for professionals working with children (nutrition educators and other professionals). This is because in order to reach children and promote healthy habits, we need to base our approach on scientific recommendations, taking into account the understanding, interest and assessments that children do about food. It is important to involve boys and girls in program planning so as to more accurately meet their needs and create more efficient interventions. Strategies for the promotion of healthy eating should be fostered, combining all elements identified as conditioning aspects of their intake, beyond health indications, to strengthen children’s interests with regard to food.

## Conclusions

Taken together, virtually all of the study participants established a relationship between their subjective well-being and food based on a multidimensional argument referring primarily to the issues of health, pleasure, emotions, empowerment and commensality (i.e., eating together). Therefore, it may be necessary to encourage healthy eating programs for children to improve health and well-being, especially if we consider that there are several daily opportunities to eat, and we know that eating becomes a significant moment to promote subjective well-being.

Data collection and analysis were performed meticulously in order to ensure the validity of the results. The comments by the groups varied; while some made numerous comments and important reflections, others gave fewer opinions with more superficial comments. However, overall we consider that the arguments used in the different groups coincided with the thinking of the majority of participants, indicating a shared and coherent view.

Although a quantitative approach would have allowed us to determine how important food is in children’s lives, it would not have been possible—as has been done here using discussion groups—to delve deeper into the reasons that make food more or less important for them (linked to the beliefs and attitudes they have towards food), what kind of relationship they maintain with food and with other people around food, and how they “negotiate” what they eat and in what quantity with the people in charge of their food intake.

In summary, we have seen that most of the boys and girls participating in the focus groups related food to subjective well-being, highlighting an important role of food in how they feel, mostly for physical reasons (health and growth), although they did also attribute a role to it in the emotional affectation of their well-being. That being said, in some groups there was some doubt about the existence of this relationship, the reason being that the way of living in this age group is independent from diet or eating habits.
